# Defective Leukocyte β2 Integrin Expression and Reactive Oxygen Species Production in Neonates

**DOI:** 10.3390/children9040494

**Published:** 2022-04-01

**Authors:** Irma Capolupo, Domenico Umberto De Rose, Roberto Pascone, Olivier Danhaive, Marcello Orzalesi

**Affiliations:** 1 Neonatal Intensive Care Unit, Medical and Surgical Department of Fetus, Newborn and Infant, IRCCS “Bambino Gesù” Children’s Hospital, 00165 Rome, Italy; domenico.derose@opbg.net (D.U.D.R.); morzalesi@interfree.it (M.O.); 2 Department of Pediatrics, “Sapienza” University of Rome, 00185 Rome, Italy; roberto.pascone@uniroma1.it; 3 Department of Neonatology, Cliniques Universitaires Saint Luc, 1200 Bruxelles, Belgium; olivier.danhaive@saintluc.uclouvain.be

**Keywords:** innate immunity, immunodeficiency, bacterial infections, newborns

## Abstract

Neonates are highly susceptible to bacterial infections, which represent a major source of mortality and morbidity in this age category. It is recognized that β2 integrins play a critical role in innate immunity by mediating leukocyte vascular adhesion, transmigration and bacterial phagocytosis. Therefore, we aimed to assess if the impaired immune functions seen in newborns may derive, in part, from a transient insufficient β2 integrin expression. In the present study we measured baseline lymphocyte function-associated antigen-1 (LFA-1 or CD11a/CD18), macrophage-1 antigen (MAC-1 or CD11b/CD18) and leukocyte integrin p150-95 (CD11c/CD18) expression on cord blood, and on the third day of life in a cohort of 35 healthy neonates, compared with a control group of 12 healthy adults. For any of the three β2 integrins, the expression on polymorphonuclear cells was significantly lower on cord blood than in adults and increased from birth to day 3. We also compared superoxide radical (SR) production in these neonates with 28 non-smoking adults. SR production in response to integrin stimulation by Zymosan was significantly lower at birth than in adults, and it decreased further in the third day of life. These findings suggest that innate immune impairment in newborns may be, in part, accounted for by a lower β2 integrin expression on phagocytes in the neonatal period, but also by a functional impairment of free radical production.

## 1. Introduction

At the neonatal age, bacterial infections represent a relevant factor of mortality and morbidity [1]. A critical factor that accounts for this elevated incidence of sepsis is the neonate’s reduced capacity of fighting bacteria. Compared with the adult, the granulocyte progenitor pool is quantitatively reduced, especially in preterm neonates [2]. Rolling, adhesion, and cell polarization functions of neonatal neutrophils are reduced, which account for their impaired chemotactic responses. The opsonizing capacity of neonatal serum is low [3], and therefore in vivo phagocytosis is impaired. Finally, the respiratory burst decreases rapidly under stress or during sepsis, leading to an impairment of bacterial killing [4]. In many of these processes, β2 integrins are implied. β2 integrins are membrane proteins with a dimeric structure that consist of a common β chain (CD18), non-covalently bound to a variable α subunit, CD11a to CD11c, leading to three heterodimers: CD11a/CD18 (Leukocyte Functional Antigen-1, LFA-1), CD11b/CD18 (complement receptor-3, CR-3, or macrophage-1 antigen, MAC-1), and CD11c/CD18 (complement receptor-4, CR-4, or p150/95) [5,6].

Their extracellular domain displays multiple receptor and adhesion sites, whereas the intracellular domain is connected to various cytoplasmic signaling pathways and alters several cell functions. LFA-1 and MAC-1, through their interaction with the intercellular adhesion molecule family (ICAM-1, 2 and 3), play a major role in leukocyte adhesion and emigration [7]. MAC-1 is also a key receptor for the phagocytosis of both opsonized and non-opsonized particles, through its complement iC3b binding site and its lectin-like site [8]. MAC-1 engagement by its ligands is a critical step in the activation of Nicotinamide Adenine Dinucleotide PHosphate (NADPH) oxidase (NOX), the enzyme responsible for the oxidative burst and bacterial killing in phagocytes [9]. Moreover, besides its expression on circulating phagocytes, MAC-1 is also the main integrin in the reticulo-endothelial system, hence contributing to circulating bacteria clearance in the liver and spleen [10]. Therefore, in this study we sought to analyze the surface expression and function of β2 integrins in a cohort of healthy neonates at birth and on the third day of life. LFA-1, MAC-1 and p150/95 surface expression on the leukocyte was determined by flow cytometry after the labeling of CD11a, CD11b, CD11c and CD18 by monoclonal antibodies.

Zymosan, a yeast extract mostly constituted of β-glucan and commonly used for in-vitro neutrophil and macrophage activation, has the property of activating integrins (mostly MAC-1) either through its lectin site when non-opsonized or through its complement-biding site when opsonized [11]. Therefore, we also analyzed NADPH-oxidase activation in response to opsonized and non-opsonized Zymosan particles in order to determine the functionality of integrin-triggered signaling in these patients.

## 2. Materials and Methods

### 2.1. Patients

In this pilot study, thirty-five consecutive neonates were recruited in the immediate prenatal period on the basis of gestational age, anticipated mode of delivery, the absence of anomalies on routine echographic and serologic screening tests during pregnancy, and the absence of risk factors for perinatal infection other than preterm labor. Informed parental consent was systematically obtained. A 1.5 mL blood sample was drawn from the umbilical cord at birth, and a second one from a venous puncture on the third day of life when routine metabolic screening tests were performed. Twelve healthy 20–40-year-old adults were recruited as controls for β2 integrin expression, and twenty-eight 20–40-year-old non-smoking patients were recruited as controls for superoxide radical production.

### 2.2. Integrin Cell Surface Expression

For each patient, 100 μL whole blood aliquots EDTA samples were drawn at birth and at the third day of life. They were processed within 6 h and diluted in 100 μL of phosphate-buffered saline (PBS). Samples were thus incubated on ice for 30 min after red cell lysis, adding 10 μL of one of the following monoclonal antibodies: fluorescein (FITC)-conjugated anti-CD11a (Agilent Dako, Santa Clara, CA, USA), anti CD11b (Ortho Diagnostic Systems, Milan, Italy), anti-CD11c (Agilent Dako, Santa Clara, CA, USA), anti-CD18 (Agilent Dako, Santa Clara, CA, USA) and FITC-conjugated anti-murine IgG (Ortho Diagnostic Systems, Milan, Italy) as a secondary antibody for CD11b and CD18 or isotype negative control. They were then analyzed with a BD FACS flow cytometer and Lysis II software (Becton-Dickinson, Franklin Lakes, NJ, USA). For each marker, the percentage of fluorescent cells was determined in the three cell populations identified by light scattering (neutrophils, monocytes and lymphocytes).

### 2.3. Superoxide Radical Production

For each patient at each time point, an 800 μL sample of heparinized whole blood was processed within 6 h from puncture in order to evaluate neutrophil superoxide radical production in resting or stimulated conditions, as previously described [12]. Briefly, the sample was divided in eight 100 µL aliquots, diluted in 0.35 mL of Krebs-Ringer-Phosphate buffer (pH 7.4) with 0.5 mMol CaCl2 and 5 mMol glucose, and exposed for 5 min to one of the four following conditions: (1) 50 µL of non-opsonized Zymosan (Z) particles (Sigma Chemical Company, St. Louis, MO, USA); (2) 50 µL of opsonized Zymosan (OZ) particles pre-incubated with pooled adult human serum; (3) 5 µL of phorbol-12 myristate-13 acetate (PMA) (Sigma Chemical Company, St. Louis, MO, USA); or (4) not-stimulated resting condition (negative control). The aliquots were then incubated at 37.0 °C for 15 min with Fe^3+^ cytochrome C, 50 µL. The reaction was then blocked with 2 µL N-ethylmaleinide (Sigma-Aldrich Corporation, St. Louis, MO, USA), the aliquots were centrifuged at 1500× *g* for 10 min, and the supernatant’s light absorbance was analyzed by spectrophotometry at the wavelengths of 468 and 550 nM. Negative controls were obtained by adding 10 µL superoxide dismutase (Sigma-Aldrich Corporation, St. Louis, MO, USA) prior to Fe^3+^ cytochrome C. The amount of superoxide radical produced by neutrophils was calculated from the product of total superoxide production value, as determined by spectrophotometry and the neutrophil fraction measured on a routine white cell differential count.

### 2.4. Statistical Analysis

Data are presented as numbers and percentages for categorical variables. Continuous variables are expressed as mean ± standard deviation (SD) if they were normally distributed. A paired sample *t*-test was used to compare the two populations’ means (“at birth” versus “at three days of life”); a two-tailed test was used to perform analysis within groups (adults–neonates and neonates born via different modalities). Statistical analyses were performed with SPSS 17.0 for Windows (SPSS, Chicago, IL, USA). A *p*-value < 0.05 was considered as significant.

## 3. Results

Samples of cord blood at birth and venous blood at 3 days of life were collected from 11 term neonates delivered vaginally, 12 term neonates delivered via cesarean section, and 12 preterm neonates (gestational age: 32–36 weeks) delivered via cesarean section. β2 integrin expression on neutrophils, monocytes and lymphocytes in neonates (on day 0 and day 3) and in adults is reported in Table 1.

**Figure 1:**
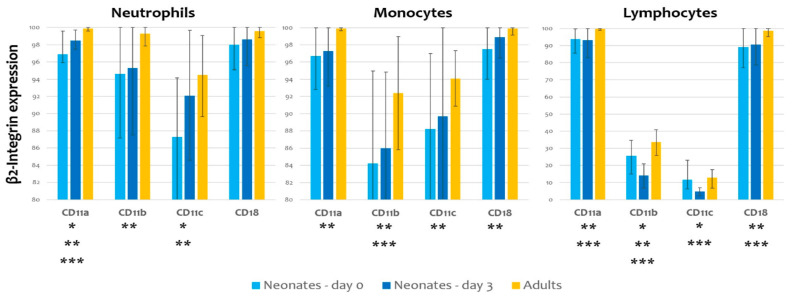
β2 integrin expression on neutrophils, monocytes and lymphocytes in neonates (on day 0 and day 3) and in adults; *p*-value < 0.05: * Neonates day 0 vs. Neonates day 3, ** Neonates day 0 vs. Adults, *** Neonates day 3 vs. Adults.

Neutrophils broadly expressed CD11a, CD11b and CD11c at birth. We observed a statistically significant raise from birth to day 3 for CD11a and CD11c (Figure 1). The monocytes’ β2 integrin expression pattern was similar, with a trend to increase from birth to day 3 and a statistically significant increase from birth to adulthood. Lymphocytes predominantly expressed CD11b and CD11c, which increased significantly from birth to day 3.

The delivery mode (vaginal delivery or caesarean section) or the gestational age (term and preterm infants) seemed to have no influence on β2 integrin expression, with no significant differences.

We also compared superoxide radical (SR) production in these neonates with 28 non-smoking adults (Table 2). We found no differences related to delivery mode (vaginal delivery or caesarean section) and gestational age (term or preterm neonates).

Whereas adults reached a near-maximal NADPH induction either after naked (Z) or opsonized zymosan (OZ) (18- to 26-fold induction from resting condition), neutrophils at birth showed a markedly impaired response to Z when compared to adults (4-fold vs. 18-fold, *p* < 0.01). Similarly, after PMA stimulation, neonates at birth had a lower SR production when compared to adults (16-fold vs. 20-fold, *p* < 0.01). Neonates at day 3 showed intermediary values.

The neonatal response to OZ at birth was much higher than that to Z (13-fold vs. 4-fold, *p* < 0.01) but still appeared blunted when compared to the stimulation obtained in adults, although not significantly (13-fold vs. 26-fold, *p* = 0.30).

At day 3, neonates showed a further decrease in NADPH inducibility after Z (2.6-fold vs. 4-fold at day 0, *p* < 0.01). The opsonization of zymosan partially restored the NADPH response in neonates at day 3, but it remained lower than the maximal response obtained with PMA (*p* = 0.02) than that displayed by adults (*p* = 0.02) and that detected in neonates at birth (*p* = 0.06).

## 4. Discussion

Herein, we assessed whether a deficit in the expression or function of β2 integrins might represent a common mechanism for the impaired innate immunity observed in newborns [13]. Yektaei-Karin et al. previously described that the CD11b expression of neutrophils in newborns was lower than in adults, but was not affected by delivery stress. They also found that neutrophils from neonates delivered vaginally had a higher transmigration ability compared with neutrophils from neonates born via cesarean section or adults, both spontaneously and after interleukin-8 (IL-8) induction. IL-8 produced during labor, associated with a complex neuroendocrine immunomodulatory pathway, would reflect cell activation and result in an enhanced chemotaxis [14].

Stålhammar et al. also observed differences between the cord blood from 16 healthy neonates and the peripheral blood from 17 healthy adults, induced by IL-8, suggesting that the neutrophil response to intermediate chemoattractants might lead to a compromised infectious response in newborn infants [15].

Our study showed that neonatal phagocytes in vitro had a lower production of superoxide radical than those of adults after incubation with Zymosan (a reagent prepared from the yeast cell wall commonly used to induce experimental sterile inflammation); hence, supposedly, a reduced bacterial killing capacity. This defective response did not appear to be related to a minor capacity of synthesis.

Indeed, after the direct activation of NADPH (the enzyme responsible for superoxide generation in phagocytes) by PMA, neonatal neutrophils showed a response actually greater than in adults. These in vitro findings suggest that the signaling pathway of NADPH activation can be less active in newborns.

β2 integrins, particularly MAC-1 and LFA-1, are known to play a critical role in phagocyte activation during sepsis [16]. We quantified β2 integrin surface expression on circulating leukocytes by flow cytometry and found that both LFA-1 and MAC-1 were broadly expressed on neutrophils and macrophages since birth. Even if we found a statistically significant, age-related increase in expression between birth, day 3, and adults, its magnitude was so small that it is unlikely to account for the profound superoxide production deficiency we observed in neonates.

Similarly, O’Hare et al. reported an increase in neutrophil CD11b expression in a cohort of preterm infants over the first week of life, but they observed a robust reactive oxygen intermediate (ROI) production after lipopolysaccharide (LPS) stimulation [17]. However, neonatal blood was obtained postnatally in their study, whereas in our cohort analyses were performed on umbilical cord blood, in addition to the differences related to the compound that was used.

Zymosan, the compound we used for leukocyte stimulation, as demonstrated in the literature [18], is mostly constituted of β-glucan, a class of polysaccharides found in bacteria and yeasts. In vivo, β-glucans activate the complement cascade and bind to the soluble complement fraction iC3b, one of the ligands of MAC-1. This interaction takes place on the I-domain of CD11b and triggers several intracellular pathways that lead to cytoskeleton activation, phagocytosis and NADPH activation through the phosphokinase C (PKC) pathway [19,20]. This process is known as complement-dependent, type II opsonic phagocytosis [21]. Additionally, β-glucans have the property of activating phagocytes through a non-opsonic pathway, through receptors known as lectins [22]. MAC-1, that carries a lectin-like epitope outside its I-domain, is considered to play a major role in type I, non-opsonic phagocytosis in macrophages and neutrophils [8].

Moreover, β-glucan binding of this lectin site activates MAC-1 and generates a cytotoxic response in macrophages and natural killer lymphocytes towards opsonized target cells [23].

The deficient oxidative burst we observed at birth after naked zymosan was mostly restored after pre-incubation of blood samples with adult human serum. This finding may be related either to a decreased opsonizing capacity of neonatal versus adult serum [3], or a minor affinity of the MAC-1 lectin site in newborns [24]. Defective opsonizing capacity in neonates has been reported in the literature, despite normal levels of complement [4,25].

We observed a further depression of superoxide production on day 3 that was significant after naked zymosan, and close to being significant after opsonized zymosan. This difference in NADPH activity between cord blood and venous blood at day 3 might be interpreted as a primed state of phagocytes at birth, returning to baseline at day 3.

In a detailed analysis of the NADPH oxidase activity and its components from unstimulated neutrophils isolated from cord blood in neonates, Chudgar et al. demonstrated a lower amount of cytosolic components, which could contribute to the impaired neutrophil response [26].

An alternative explanation is the presence of high serum levels of anti-oxidant substances such as bilirubin or carbon monoxide (CO), known to have immunomodulator properties, during the first days of life [27]. It is possible that these products mediate the reduced oxidative burst capacity that we observed [27]. Indeed, the polymorphisms identified in this pathway (in the gene encoding heme oxygenase—HO) seem to contribute to neonatal jaundice and other perinatal complications such as bronchopulmonary dysplasia [28]. Furthermore, bilirubin has been shown to decrease leukocyte vascular adhesion under oxidant stress [29], while hemin (a HO substrate analog) has been demonstrated to inhibit the cell surface expression of CD11b and CD66b on human neutrophils [30].

Morisaki et al. also determined in rats that endogenous CO attenuates endotoxin-induced adhesive responses of platelets and contributes to the amelioration of leukocyte adhesion in venules [31]. Moreover, CO acts a down-regulator of several pro-inflammatory genes, and this has been also confirmed by studies on fetal human membranes, where its role seemed to promote an anti-inflammatory environment during intrauterine infections by inhibiting Tumor Necrosis Factor α (TNF-α) and interleukin 1β (IL-1β) production [32].

Limitations of this study include the lack of standardized inclusion criteria, and the small sample size, for whom we probably could not highlight differences related to gestational age or delivery mode.

On the other hand, to know the normal values of these markers in healthy neonates is crucial, considering recent studies about neutrophil CD11b during infectious episodes that indicate that it could be a promising biomarker for the early diagnosis of neonatal sepsis [33,34].

## 5. Conclusions

Innate immunity plays a key role in neonatal responses, although a decreased expression level of adhesion molecules on white blood cells and impaired phagocytosis and opsonization. Our findings suggest that innate immune impairment in newborns may be, in part, accounted for by a lower β2 integrin expression on phagocytes in the neonatal period, but also by a functional impairment of free radical production. However, further studies are needed to understand if these in vitro findings are clinically relevant in vivo and play a role in susceptibility to neonatal sepsis.

## Figures and Tables

**Table 1:** β2 integrin expression on neutrophils, monocytes and lymphocytes in neonates (on day 0 and day 3) and in adults.

		Neonates—Day 0	Neonates—Day 3	Adults	*p*-Value (Neonates Day 0 vs. Day 3)	*p*-Value (Neonates Day 0 vs. Adults)	*p*-Value (Neonates Day 3 vs. Adults)
Neutrophils	CD11a	96.9 ± 2.7	98.5 ± 1.3	99.9 ± 0.1	<0.01 *	<0.01 *	<0.01 *
	CD11b	94.6 ± 7.5	95.3 ± 7.6	99.3 ± 1.4	0.70	0.04 *	0.08
	CD11c	87.3 ± 7.0	92.1 ± 7.6	94.5 ± 4.7	<0.01 *	<0.01 *	0.31
	CD18	98.0 ± 2.7	98.6 ± 2.9	99.6 ± 1.1	0.37	0.05	0.25
Monocytes	CD11a	96.7 ± 4.0	97.3 ± 4.3	99.9 ± 0.1	0.55	<0.01 *	0.12
	CD11b	84.2 ± 10.9	86.0 ± 9.0	92.4 ± 6.5	0.45	0.02 *	0.03 *
	CD11c	88.2 ± 8.9	89.7 ± 10.6	94.1 ± 3.4	0.52	0.03 *	0.17
	CD18	97.5 ± 3.5	98.9 ± 2.5	99.9 ± 0.2	0.06	0.02 *	0.18
Lymphocytes	CD11a	93.8 ± 7.0	93.1 ± 10.2	99.8 ± 0.3	0.74	<0.01 *	0.03 *
	CD11b	25.5 ± 9.9	14.1 ± 6.6	33.6 ± 7.5	<0.01 *	<0.01 *	<0.01 *
	CD11c	11.5 ± 6.0	4.8 ± 2.6	12.8 ± 5.5	<0.01 *	0.51	<0.01 *
	CD18	89.2 ± 12.3	90.7 ± 11.4	98.6 ± 4.6	0.60	0.01 *	0.03 *

* *p*-value < 0.05 are statistically significant.

**Table 2:** Superoxide radical (SR) production in neonates (on day 0 and day 3) and in non-smoking adults.

	Resting	After Zymosan (Z)	After Opsonized Zymosan (OZ)	After PMA
Day 0	4.39 ± 4.00	18.42 ± 17.56	59.53 ± 27.77	72.99 ± 29.06
Day 3	2.28 ± 3.58	6.22 ± 10.97	47.80 ± 22.97	63.12 ± 30.27
Adults	2.61 ± 1.73	48.78 ± 34.20	69.02 ± 43.11	52.21 ± 28.66

## Data Availability

The original contributions presented in the study are included in the article; further inquiries can be directed to the corresponding author.
